# Iatrogenic Spinal Cord Injury during Removal of the Inferior Articular Process in the Presence of Ossification of the Ligamentum Flavum

**DOI:** 10.1155/2016/2318759

**Published:** 2016-01-18

**Authors:** Shane M. Burke, Steven W. Hwang, Mina G. Safain, Ron I. Riesenburger

**Affiliations:** ^1^Department of Neurosurgery, Tufts Medical Center, Boston, MA 02111, USA; ^2^Department of Neurosurgery, Tufts University School of Medicine, Boston, MA 02111, USA

## Abstract

Ossified ligamentum flavum (OLF) is a condition of heterotopic lamellar bone formation within the yellow ligament. Some patients with OLF can be asymptomatic. However, asymptomatic OLF may not be obvious on preoperative MRI and could increase the risk of iatrogenic injury during treatments for unrelated spinal conditions. This report describes a case of spinal cord injury caused by the indirect transmission of force from an osteotome to an asymptomatic OLF during the resection of a thoracic inferior articular process (IAP). To prevent this outcome, we urge careful review of CT imaging in the preoperative setting and advocate the use of a high-speed drill instead of an osteotome during bone removal in the setting of an adjacent area of OLF.

## 1. Introduction

Ossified ligamentum flavum (OLF) is a condition of heterotopic lamellar bone formation within the yellow ligament [1]. Current models propose that this ossification is the net result of mechanical stresses [2] and biochemical [3, 4] and hereditary factors [5]. It is well known that OLF can cause myelopathy as the lesion grows, which may require surgical management [6–11].

However, some patients with OLF can be asymptomatic [12]. Those harboring subclinical OLF are not immune to coexisting spinal conditions such as foraminal stenosis or scoliosis [13]. Indeed, such degenerative conditions frequently coexist with OLF. Asymptomatic OLF may increase the risk of iatrogenic injury during treatments for adjacent disease. To our knowledge, this has not been reported.

Resection of a thoracic inferior articular process (IAP) is frequently performed to expose the thoracic pedicle screw entry site during preparation for screw placement. The IAP is traditionally removed with either an osteotome or a high-speed drill. We report an iatrogenic spinal cord injury caused by indirect transmission of force from an osteotome to a previously asymptomatic OLF while resecting a thoracic IAP.

## 2. Case Presentation

### 2.1. History and Examination

**Figure 1:**
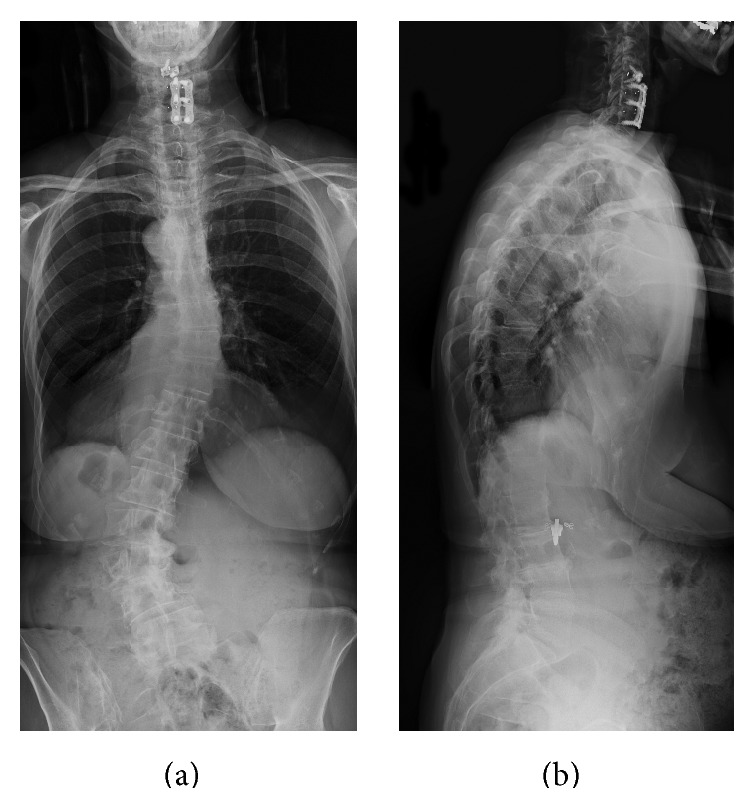
Posterior-anterior (a) and lateral (b) preoperative 3-foot scoliosis X-rays. Note the prominent curvature of the lumbar spine, which resulted in progressive symptoms.

A 71-year-old Caucasian woman with symptomatic kyphoscoliosis and multilevel lumbar spinal stenosis had been followed up in our clinic for sagittal imbalance and fatigue with progressive low-back and leg pain as well as subjective weakness of the legs bilaterally (Figure 1). Physical therapy and epidural steroid injections failed to improve her symptoms. Her strength and reflexes were normal in the lower extremities. She was elected for surgery after conservative measures failed to improve her symptoms.

### 2.2. Operation

We performed T10 to pelvic fixation with posterior laminectomies from L2 to S1, a pedicle subtraction osteotomy at L3 to correct her sagittal imbalance, and transforaminal lumbar interbody fusion at L5-S1 to decrease the L5-S1 pseudarthrosis rate. Initial neurophysiologic monitoring demonstrated consistently robust responses. An osteotome and mallet were used to resect the IAPs in preparation for pedicle screw placement.

**Figure 2:**
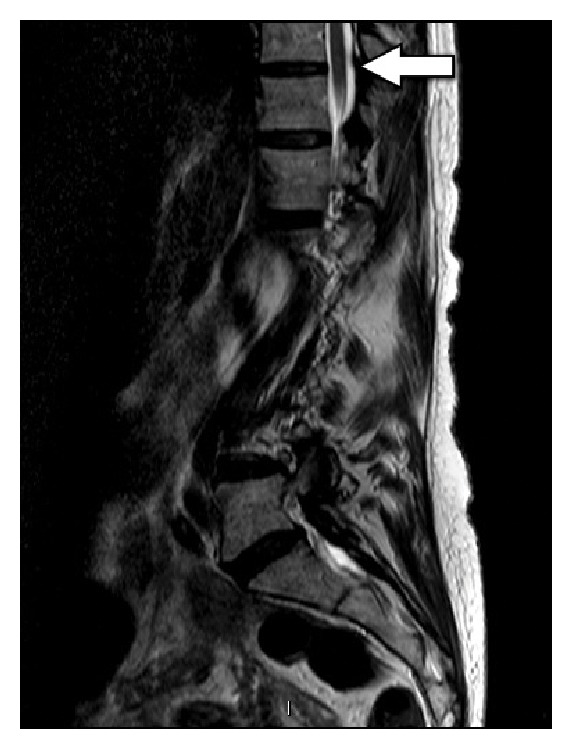
Preoperative sagittal T2 Magnetic Resonance Image. Note the absence of stenosis in the midline of the lower thoracic spine. The arrow points to the T10-11 level.

During resection of the leftT11 IAP, somatosensory evoked potentials (SSEPs) and motor evoked potentials (MEPs) were lost immediately after impact of the osteotome. The resection of this IAP was performed in a standard fashion and the spinal canal was not violated during this maneuver. We further inspected the area and did not find fracture of the adjacent superior articular process or lamina. The screw tracts were checked and none of the pedicle screws violated the spinal canal. We reviewed her preoperative MRI and the canal did not demonstrate clear stenosis or compression at that level (Figure 2). In an attempt to restore monitoring, her mean arterial pressure (MAP) was increased and sedation decreased. We contemplated performing a Stagnara test, but the SSEPs and MEPs improved. Given this improvement, and the difficulty in performing a Stagnara test in a geriatric patient, we elected to proceed with the operation. During closure of the PSO, the MAP was maintained above 90 and the osteotomy closed well with only minor dural buckling. The operation was further complicated by an unintended durotomy at the L3-4 level which was primarily closed.

**Figure 3:**
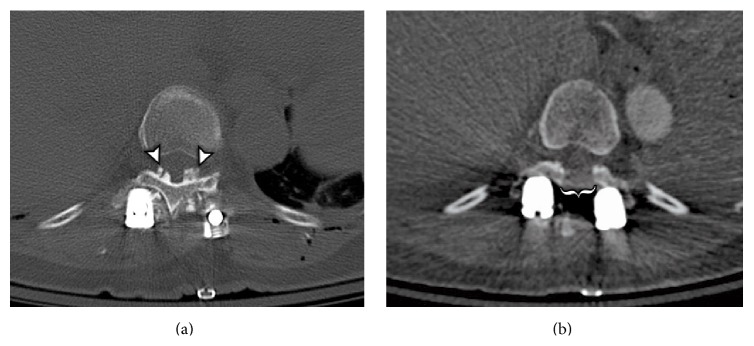
Postfacetectomy (a) and postlaminectomy (b) axial Computed Tomograms at the level of T10-11. The arrowheads in (a) demonstrate bilateral ossifications of the ligamentum flavum occupying the posterior aspect of the spinal canal. The bracket in (b) indicates the area where decompression was achieved via laminectomy and resection of these lesions.

Postoperatively, the left leg was plegic with 2/5 strength in the right leg. While still intubated, we transported her to the computed tomography (CT) scanner. Thoracic CT scans demonstrated a preexisting area of hypertrophic bone not obvious on the MRI which was reviewed intraoperatively (Figure 3(a)). Bilateral osteophytes, consistent with OLF, were occupying the canal at the T10-11 level. The osteophyte on the left was larger than the one on the right. We felt the compression secondary to the OLF was likely aggravated by the use of the osteotome during the SPO. Therefore, we returned to the OR for emergent decompression. This was achieved with a T10-11 laminectomy and bilateral OLF resection with the M8 drill and Kerrison instruments (Figure 3(b)). After the laminectomy, strength was 4/5 in the right leg, 2/5 in the proximal left leg, and 4/5 in the distal left leg.

### 2.3. Postoperative Course

Her postoperative course was complicated by a pulmonary embolism on postoperative day (POD) three, which required systemic anticoagulation. Her neurologic exam improved minimally prior to discharge on POD 10, and she was still unable to independently ambulate at that time. On POD 15, the patient returned with cerebral spinal fluid (CSF) leaking through the caudal aspect of the incision. She required two additional operations and temporary CSF diversion via a lumbar subarachnoid drain to stop the leak. By POD 32, the incision was fully healed and she had no residual signs or symptoms of persistent CSF leakage. Her postoperative course was further complicated by a non-ST elevation myocardial infarction treated with aspirin and beta blockers. She was discharged to a rehabilitation facility on POD 40.

**Figure 4:**
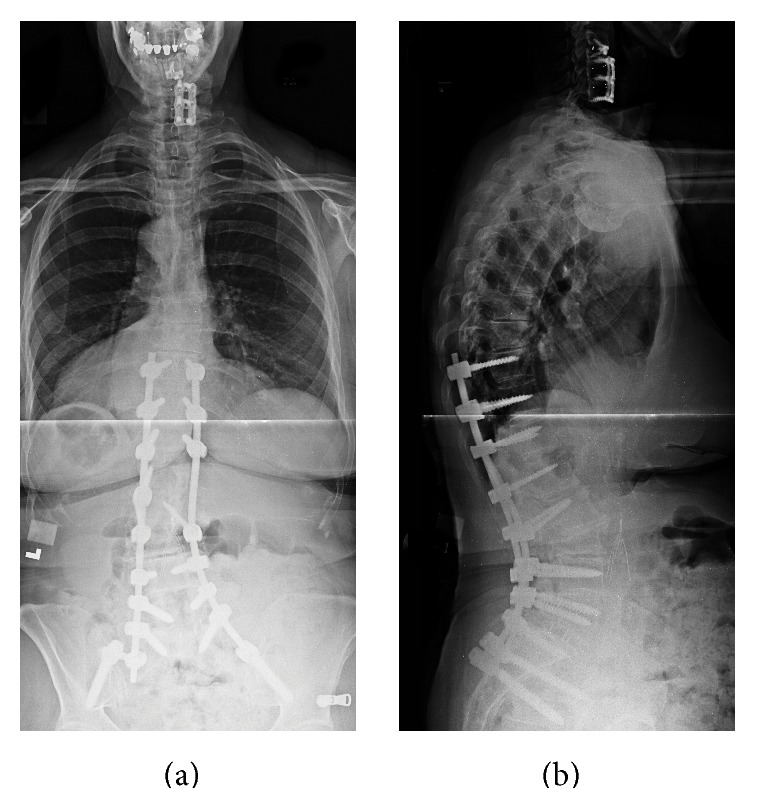
Posterior-anterior (a) and lateral (b) postoperative 3-foot scoliosis X-rays. Note the markedly improved coronal and sagittal balance achieved after the deformity correction.

Two months postoperatively, she had full strength and was able to walk independently. However, she had some residual lower extremity hyperreflexia secondary to the intraoperative spinal cord injury. She reported that while her preoperative symptoms were not completely resolved, they were improved. She continues to ambulate independently upon one-year follow-up, with no radiographic evidence of hardware failure (Figure 4).

## 3. Discussion

While several case series cite higher rates of nerve root and spinal cord injury (30%) during deformity correction surgery [14–16], most of these papers were published prior to the development of modern techniques [17–19]. Similarly, although outcomes after decompressive procedures for myelopathic OLF patients are mixed (10–30% of patients experience neurological deficits after decompression for ossification of the posterior longitudinal ligament) [20–23], no cases of iatrogenic injury due to asymptomatic OLF have been previously reported. The preceding case reflects a mechanism by which an incidental OLF facilitated iatrogenic injury secondary to correction of a different degenerative condition, which to our knowledge has not been previously reported. We performed a review of the literature to evaluate the pertinent aspects of this case and suggest general recommendations intended to minimize such incidents in the future.

The clinical presentation of OLF is dependent on the region of compression. Myelopathy is by far the most common presentation since most lesions occur in the thoracic and cervical regions [1, 9, 24–26]. Our subject did not have a myelopathic exam preoperatively, thus confirming she was asymptomatic from her OLF. A handful of cases have also described radiculopathy as a diagnostic possibility of OLF, although this was not observed in our case and is quite rare [26, 27].

The measured prevalence of OLF varies based on the selected imaging modality and study population. One cross-sectional study that analyzed thoracic CT scans from Chinese patients admitted for chest pain reported an OLF prevalence rate of 63.9% [28]. An American study that analyzed CT scans obtained from a similar patient population reported an OLF prevalence rate of 26% [29], while a study that analyzed MRI instead of CT scans reported an OLF prevalence rate of only 3.8% [12]. These data demonstrate that CT is a more sensitive imaging modality for detecting OLF and highlight a higher prevalence in Asian populations. These differential prevalence rates suggest that OLF may often be asymptomatic and underreported in those only receiving MRIs. Thus, the mechanism of injury from our case may be relevant to a large number of patients.

This case demonstrated a calcified mass that was not clearly visible on preoperative MRI but could be observed on CT. The evolution of these lesions explains the differing sensitivities of various imaging modalities. OLF usually starts laterally, in the capsular portion of the ligamentum flavum, and then spreads to involve the medially located, interlaminar portion [30]. At this stage, the ossification only represents a thin strip of bone that may not be discernible from adjacent structures on MRI [28]. In such instances, CT has been shown to be more sensitive for resolving the pathology because the appearance of a hyperdense osteophyte is more obvious than the hypointense appearance on MRI [28]. Without cause to take notice, we did not appreciate the area of OLF when we reviewed her CT scan to plan our osteotomies and pedicle screw trajectories. Furthermore, when SSEPs and MEPs were lost intraoperatively, we reviewed the preoperative MRI but did not review the CT. On the MRI, we did not appreciate the area of OLF. Had we had reviewed the preoperative CT after losing SSEPs and MEPs, we would have recognized the OLF and immediately performed a decompressive laminectomy and OLF resection, without having to take the patient back to the operating room a second time. Thus, we recommend that surgeons review both preoperative MRI and CT scans when MEPs and SSEPs are lost intraoperatively.

The theorized mechanism of injury during this case involved the transmission of force from the osteotome to the OLF. In order to resect the T11 IAP, the osteotome was initially directed in a rostral orientation. We propose that when the IAP was struck with the osteotome, the force vector was also directed rostrally through the OLF, which resided at the T10-11 level. This subsequently resulted in contusion of the spinal cord adjacent to the OLF. Therefore, we strongly suggest that surgeons consider using a high-speed drill rather than an osteotome when removing bone adjacent to areas of stenosis such as OLF, even if these areas are asymptomatic preoperatively.

To avoid the possibility of iatrogenic injury due to an asymptomatic OLF, it is critical to entertain OLF as a diagnostic possibility, even in asymptomatic patients, when reviewing preoperative films. Since CT is more sensitive and specific for these lesions, we recommend careful scrutiny of preoperative CT scans. Lastly, this incident could have been avoided by electing to use a high-speed drill in lieu of an osteotome to resect the IAP. In light of this case, we feel the use of a high-speed drill should be strongly considered when resecting bone in areas adjacent to an OLF. We have modified our management to reflect these recommendations without further incident.
